# Photoimmunotheranostic agents for triple-negative breast cancer diagnosis and therapy that can be activated on demand

**DOI:** 10.18632/oncotarget.10705

**Published:** 2016-07-19

**Authors:** Manal Amoury, Dirk Bauerschlag, Felix Zeppernick, Verena von Felbert, Nina Berges, Stefano Di Fiore, Isabell Mintert, Andreas Bleilevens, Nicolai Maass, Karen Bräutigam, Ivo Meinhold-Heerlein, Elmar Stickeler, Stefan Barth, Rainer Fischer, Ahmad Fawzi Hussain

**Affiliations:** ^1^ Department of Experimental Medicine and Immunotherapy, Institute of Applied Medical Engineering, Helmholtz-Institute for Biomedical Engineering, 52074 Aachen, Germany; ^2^ Department of Gynecology and Obstetrics, University Medical Center Schleswig-Holstein, 24105 Kiel, Germany; ^3^ Department of Gynecology and Obstetrics, University Hospital RWTH Aachen, 52074 Aachen, Germany; ^4^ Department of Dermatology, University Hospital RWTH Aachen, 52074 Aachen, Germany; ^5^ Fraunhofer Institute for Molecular Biology and Applied Ecology IME, 52074 Aachen, Germany; ^6^ Department of Nuclear Medicine, University Hospital RWTH Aachen, 52074 Aachen, Germany; ^7^ Department of Gynecology and Obstetrics, University Hospital Schleswig-Holstein, 23538 Lübeck, Germany; ^8^ Department of Pharmaceutical Product Development, Fraunhofer Institute for Molecular Biology and Applied Ecology IME, 52074 Aachen, Germany; ^9^ Current address: Institute of Infectious Disease and Molecular Medicine (IDM), Department of Integrative Biomedical Sciences, Faculty of Health Sciences, University of Cape Town, Cape Town 7925, South Africa; ^10^ Institute of Molecular Biotechnology, RWTH Aachen University, 52074 Aachen, Germany

**Keywords:** breast cancer, theranostics, photodynamic therapy, molecular targeting, antibody-based therapy

## Abstract

Triple-negative breast cancer (TNBC) is a heterogeneous disease in which the tumors do not express estrogen receptor (ER), progesterone receptor (PgR) or human epidermal growth factor receptor 2 (HER2). Classical receptor-targeted therapies such as tamoxifen or trastuzumab are therefore unsuitable and combinations of surgery, chemotherapy and/or radiotherapy are required. Photoimmunotheranostics is a minimally invasive approach in which antibodies deliver nontoxic photosensitizers that emit light to facilitate diagnosis and produce cytotoxic reactive oxygen species to induce apoptosis and/or necrosis in cancer cells. We developed a panel of photoimmunotheranostic agents against three TNBC-associated cell surface antigens. Antibodies against epidermal growth factor receptor (EGFR), epithelial cell adhesion molecule (EpCAM) and chondroitin sulfate proteoglycan 4 (CSPG4) were conjugated to the highly potent near-infrared imaging agent/photosensitizer IRDye^®^700DX phthalocyanine using SNAP-tag technology achieving clear imaging in both breast cancer cell lines and human biopsies and highly potent phototherapeutic activity with IC50values of 62–165 nM against five different cell lines expressing different levels of EGFR, EpCAM and CSPG4. A combination of all three reagents increased the therapeutic activity against TNBC cells by up to 40%.

## INTRODUCTION

Despite recent reduction in breast cancer mortality, breast cancer remains the leading cause of women's cancer death worldwide [1]. The molecular basis of breast cancer is well understood and five molecular subtypes are recognized: luminal A and B, HER2^+^, basal-like and normal breast-like [2]. Targeted therapies that rely on the expression of the estrogen receptor (ER), progesterone receptor (PgR) and epidermal growth factor receptor 2 (HER2) are effective treatments for luminal and HER2^+^ breast cancers [3].

Triple-negative breast cancer (TNBC) is a heterogeneous disease characterized by the absence of ER, PgR and HER2, and up to six distinct biological subgroups are reported. TNBC is more common in premenopausal patients, accounting for up to 20% of breast cancer cases [4]. TNBC has a generally poor prognosis with lower disease-free and overall survival [5] when compared to luminal tumors. TNBC patients do not benefit from drugs such as tamoxifen, aromatase inhibitors, trastuzumab, pertuzumab, trastuzumab-emtansin (TDM- 1) or palbociclib, which target the absent receptors. Treatment therefore relies on surgery, chemotherapy and/or radiotherapy.

Targeted therapy is a successful paradigm in oncology, increasing both efficacy and safety. Antibody-drug conjugates (ADC) are promising tumor-targeting drugs that selectively eliminate cancer cells by combining the receptor-targeting specificity of a monoclonal antibody (mAb) with a highly potent cytotoxic agent [6]. The first-in-class ADC for breast cancer treatment is TDM-1, which combines trastuzumab and emtansine and is highly effective against recurrent HER2^+^ breast cancer, even after trastuzumab therapy [7]. However, the cytotoxic agents in ADCs are typically conjugated randomly to the antibodies using either the reduced sulfhydryl groups of cysteine residues, or the amino groups of lysine side chains. This generates heterogeneous ADC populations with variable drug-to-antibody ratios (DARs) [8]. Alternative strategies for drug conjugation that generate homogeneous ADCs can significantly improve efficacy, pharmacokinetic properties and tolerability.

Although several site-specific conjugation methods have been developed to generate ADCs with defined pharmacokinetic properties, therapeutic indices, and safety profiles, their complexity and high production costs are significant drawbacks [9]. Self-labeling proteins such as the SNAP-tag provide an elegant solution to these challenges. The SNAP-tag is a derivative of the human O^6^-alkylguanine-DNA alkyltransferase (AGT) which has the ability to conjugate benzylguanine (BG) molecules depending on its folding pattern [10, 11]. SNAP-tag technology is ideally suited for protein modification and allows a fusion protein to be equipped with many different chemical entities under physiological conditions with high efficacy and a 1:1 stoichiometry. This technology has been applied in diverse experimental systems, ranging from the in-cell labeling of tagged proteins to the immobilization of proteins on chip surfaces. The SNAP-tag has also been used to generate several recombinant antibody-based fusion proteins for diagnostic and therapeutic purposes [12–15].

Another drawback of current ADCs is that constitutively active cytotoxic components must be delivered to the target cells, such that receptor binding and uptake into the cytosol are required. The ADC binds to its receptor and is internalized into lysosomes where it is degraded by lysosomal proteases, thereby releasing the cytotoxic drug into the cytosol as required to induce cell death. However, the free drug can also be released from the degraded antibody into the blood, a phenomenon known as premature drug release that can damage healthy cells. In many ADCs, cleavable linkers are used to conjugate drug molecules to the antibody in order to facilitate drug release inside the cells, which exacerbates the problem of premature release and the associated off-target toxicity [8, 16].

Theranostic agents, which combine diagnosis and therapy in one molecule, represent a promising new paradigm in cancer treatment. Most theranostics currently undergoing clinical development are nanoparticle-based reagents, which suffer drawbacks such as systemic and cellular toxicity, off-target accumulation, complexity and high production costs [17]. Photoimmunotheranostics combine the highly potent phototherapeutic activity and powerful imaging properties of photosensitizers with the specificity of mAbs or their fragments to detect and selectively eliminate tumor cells. This is a minimally invasive approach that uses nontoxic photosensitizers and harmless light, which in combination with oxygen leads to the production of cytotoxic reactive oxygen species that kill malignant cells by inducing cell apoptosis and/or necrosis [18].

The heterogeneity of TNBC limits the success of targeted treatments and it is widely acknowledged that no single therapeutic agent has the same effect on all TNBC patients. Therefore, we have developed photoimmunotheranostic agents against three receptors that are strongly expressed in TNBC: the epidermal growth factor receptor (EGFR), the epithelial cell adhesion molecule (EpCAM) and chondroitin sulfate proteoglycan 4 (CSPG4) also known as melanoma-associated chondroitin sulfate proteoglycan (MCSP) [19–21]. We generated three recombinant fusion proteins by genetically linking the SNAP-tag protein to single chain antibody fragments (scFv) targeting each receptor, namely scFv-425 (targeting EGFR), anti-EpCAM(scFv) and anti-CSPG4(scFv). The recombinant fusion proteins were conjugated with the highly potent photosensitizer, IRDye^®^700DX phthalocyanine (IR700) using SNAP-tag technology. These agents are inherently safe due to the non-toxic effect of free IR700 even after irradiation, and our results demonstrate their powerful imaging properties and potent phototherapeutic activity, individually and in combination, against four different TNBC cell lines (Hs758T, MDA-MB-231, MDA-MB-453 and MDA-MB-468) and ER^+^ breast cancer cell line (MCF-7) expressing different levels of EGFR, EpCAM and CSPG4.

Our study combines several different approaches that allow the development of novel diagnostic and therapeutic options suitable for TNBC patients. This approach could facilitate patient pre-screening, real-time treatment monitoring and *in situ* or on-demand drug activation as well as longitudinally quantifying the efficacy of treatment.

## RESULTS

### Site-specific conjugation of the fusion proteins with BG-modified fluorophores

**Figure 1:**
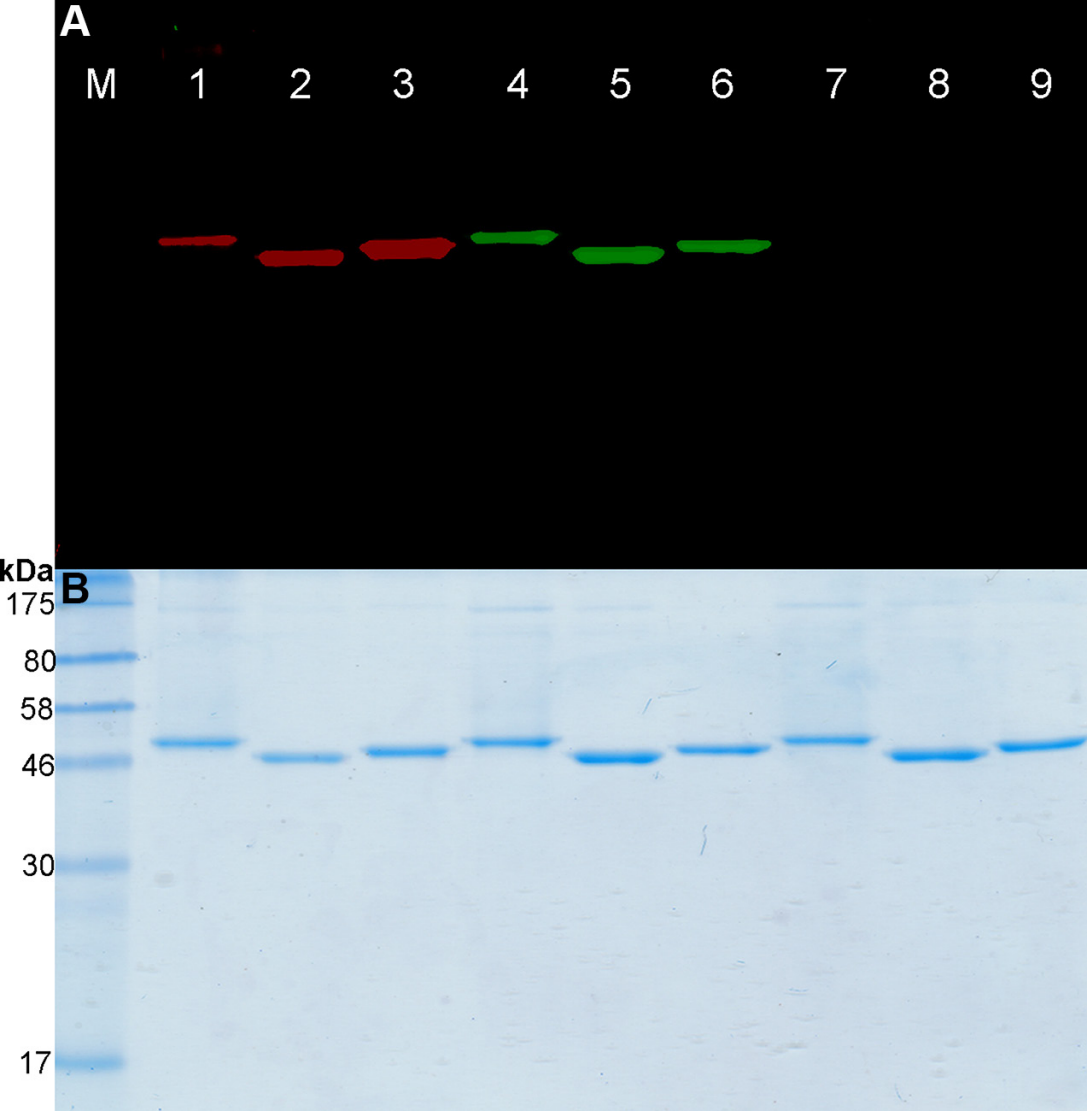
Fusion proteins labeling with BG-PEG_24_-IR700 (**A**) SDS-PAGE followed by fluorescence visualization and (**B**) subsequent Coomassie staining of BG-PEG_24_-IR700 or BG-Vista Green coupled to the SNAP-tag fusion proteins. Lanes 1–3: Fusion proteins (scFv-425-SNAP-tag, anti-EpCAM(scFv)-SNAP-tag and anti-CSPG4(scFv)-SNAP-tag) were labeled with a two-fold molar excess of BG-PEG_24_-IR700. Lanes 4–6: The same proteins were labeled with BG-Vista Green. Lanes 7–9: The SNAP-tag blocking reagent BTP was added to the fusion proteins, followed by incubation with a two-fold molar excess of BG-PEG_24_-IR700, then a two-fold molar excess of BG-Vista Green. M: Prestained Protein Marker Broad Range (7−175 kDa). Fluorescence visualization of Vista Green (green bands) and IR700 (red bands) was achieved using the CRi Maestro multispectral imaging system.

The BG-Vista Green, BG-Alexa Fluor^®^647 and BG-PEG_24_-IR700 molecules were conjugated to the three fusion proteins using the BG-SNAP-tag reaction as described above (Figure 1, lanes 1–6). The site-specific conjugation of BG-modified IR700 to the SNAP-tag fusion protein was confirmed by a BG irreversible blocking assay. The proteins were treated with bromothenylpteridine (BTP) which blocks the SNAP-tag by irreversible transfer of the alkyl group to cysteine residue. Following treatment, the coupling of the fusion proteins to BG-PEG_24_-IR700 was completely obstructed.

(Figure 1, lanes 7–9). Furthermore, we achieved a conjugation efficiency of ~90% using the BG-modified fluorophores. This was confirmed by spectrophotometry using the extinction coefficients of the fluorescence dyes and the theoretical extinction coefficient of the proteins (Table 1).

**Table 1:** Labeling efficiency of the SNAP-tag fusion proteins

	BG-Vista Green	Alexa Fluor^®^647	BG-IR700
**scFv-425-SNAP**	92	90	88
**Anti-EpCam(scFv)-SNAP**	90	90	89
**Anti-CSPG4(scFv)-SNAP**	92	89	88

Data are presented as percentage for three replicated experiments.

### Protein serum stability

**Figure 2:**
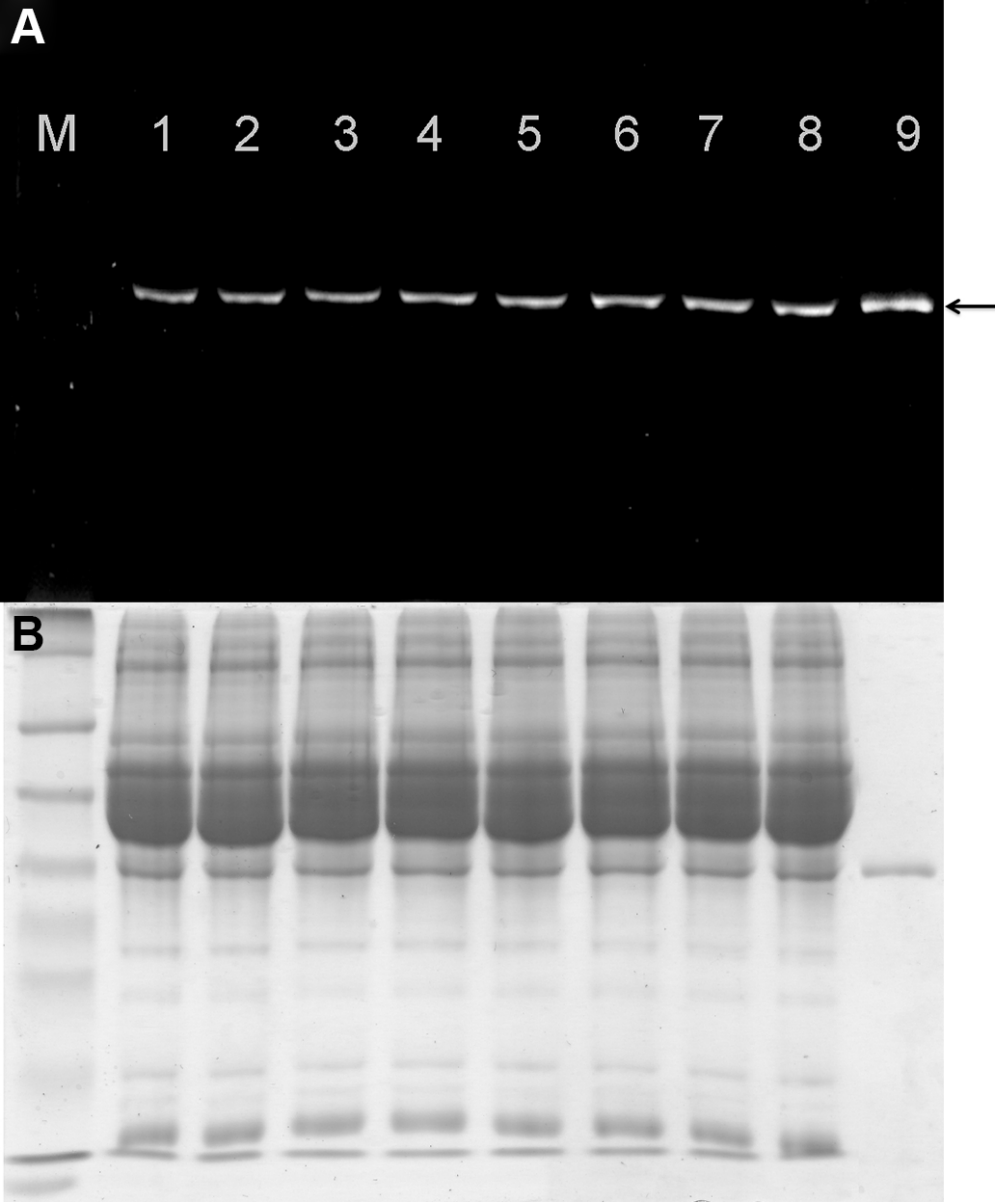
Stability of fluorescence-labeled scFv-425-SNAP protein in mouse serum (**A**) The IR700 fluorescence signal was detected using the CRi Maestro multispectral imaging system (black arrow). M: Prestained Protein Marker Broad Range (7−175 kDa). Lanes 1–8: scFv-425-SNAP-IR700 incubated with mouse serum for 0, 0.5, 1, 2, 3, 4, 5 and 6 h. Lane 9: scFv-425-SNAP-IR700 positive control. (**B**) Corresponding SDS-PAGE gel showing scFv-425-SNAP-IR700 stained with Coomassie Brilliant Blue.

The stability of the labeled proteins was analyzed using AIDA software following incubation with mouse serum for up to 6 h at 37°C. Analysis of the fluorescence signal and subsequent staining with Coomassie Brilliant Blue revealed no significant loss of the fluorescence signal (Figure 2A) or protein degradation (Figure 2B) after incubation in mouse serum for 6 h. Figure 2 shows a representative protein stability assay (scFv-425-SNAP) but similar results were observed for the anti-EpCAM(scFv)-SNAP and anti-CSPG4(scFv)-SNAP proteins.

### Flow cytometry and confocal microscopy

**Figure 3:**
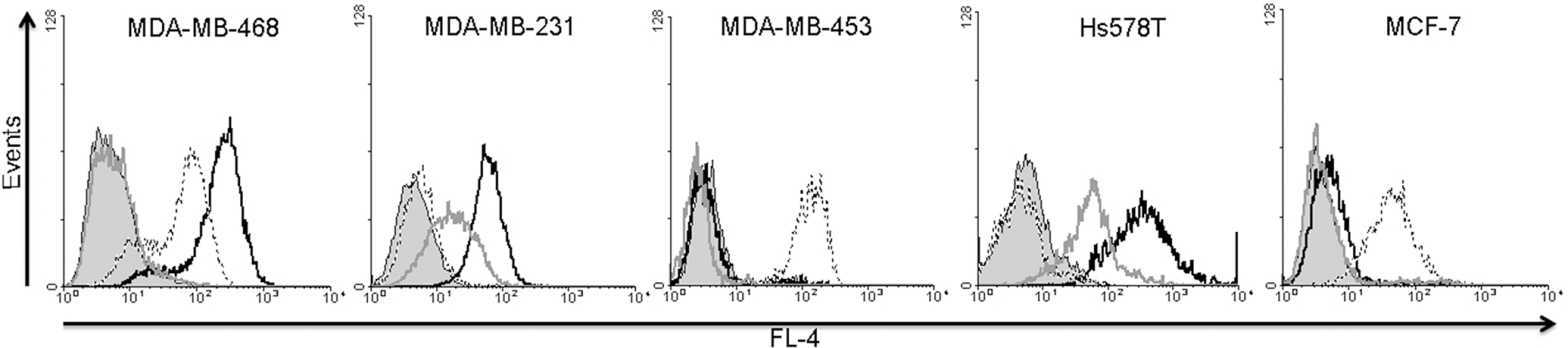
Analysis of EGFR, EpCAM and CSPG4 protein expression in MDA-MB-468, MDA-MB-231, MDA-MB-453, HS578T and MCF7 cell lines Flow cytometry was carried out after incubating the cells with Alexa Fluor^®^647-labeled scFv-SNAP fusion proteins against EGFR (black curves), EpCAM (dotted black curves) and CSPG4 (gray curves) for 30 min at 37°C in PBS. The filled gray curves represent the non-stained control.

The binding properties of the Alexa Fluor^®^647-labeled proteins was tested against four different TNBC cell lines (MDA-MB-468, MDA-MB-453, MDA-MB-231 and Hs758T) as well as the ER^+^ cell line MCF-7 by flow cytometry. Consistent with previous studies [19–21], we found that the receptors were expressed at different levels on these cell lines (Table 2 and Figure 3). MDA-MB-468, MDA-MB-453 and MCF-7 cells expressed high levels of EpCAM, whereas MDA-MB-468, MDA-MB-231 and Hs578T cells expressed high levels of EGFR. The MDAMB-231 and Hs758T cell lines expressed high levels of CSPG4. The MDA-MB-453 and MCF-7 cell lines expressed minimal levels of EGFR, and the MDA-MB-231 and Hs578T cells lines expressed minimal levels of EpCAM. Cell lines MDA-MB-468, MDA-MB-453 and MCF-7 expressed minimal levels of CSPG4 (Table 2).

**Table 2:** Expression profile for EGF, EpCAM and CSPG4 receptor in four different TNBC cell lines and ER^+^ MCF-7 cells

	EGFR	EpCAM	CSPG4
**MDA-MB-468**	+	+	−
**MDA-MB-231**	+	−	+
**MDA-MB-453**	−	+	−
**Hs758T**	+	−	+
**MCF-7**	−	+	−

**Figure 4:**
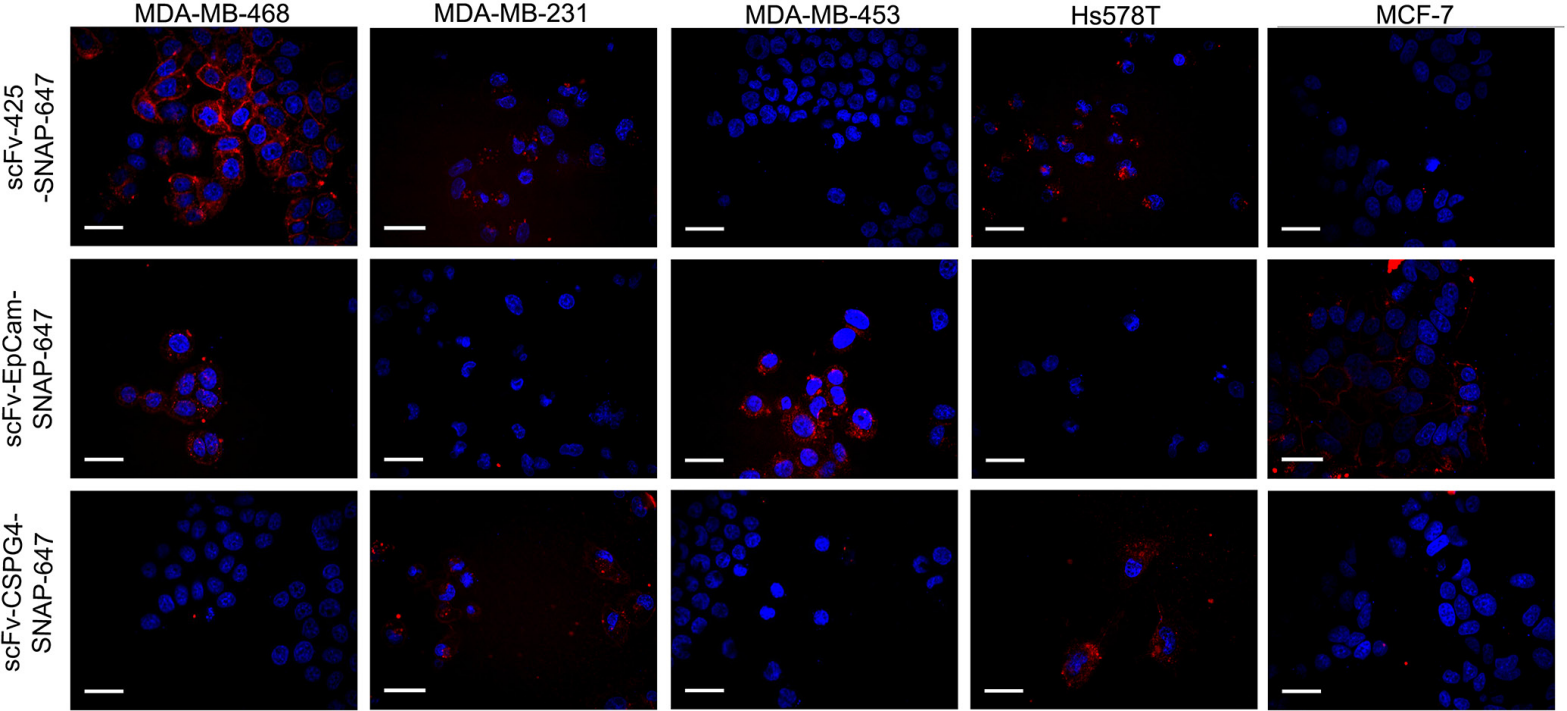
Confocal microscopy showing fluorescence-labeled scFv-SNAP fusion proteins targeting EGFR, EpCAM and CSPG4 The cells were incubated with 0.5 μg of each labeled scFv-SNAP-tag fusion protein (red signal) for 60 min at 37°C and then with Hoechst 33342 nuclear counterstain (blue signal).

The binding of the labeled fusion proteins to the cell lines was confirmed by automated confocal microscopy. The breast cancer cell lines were incubated with Alexa Fluor^®^ 647- labeled fusion proteins at 37°C. The differential staining of the cell membrane and the cellular uptake of the fusion proteins corresponded to the expression levels of EGFR, EpCAM and CSPG4 (Figure 4).

### Binding of fusion proteins to human samples

**Figure 5:**
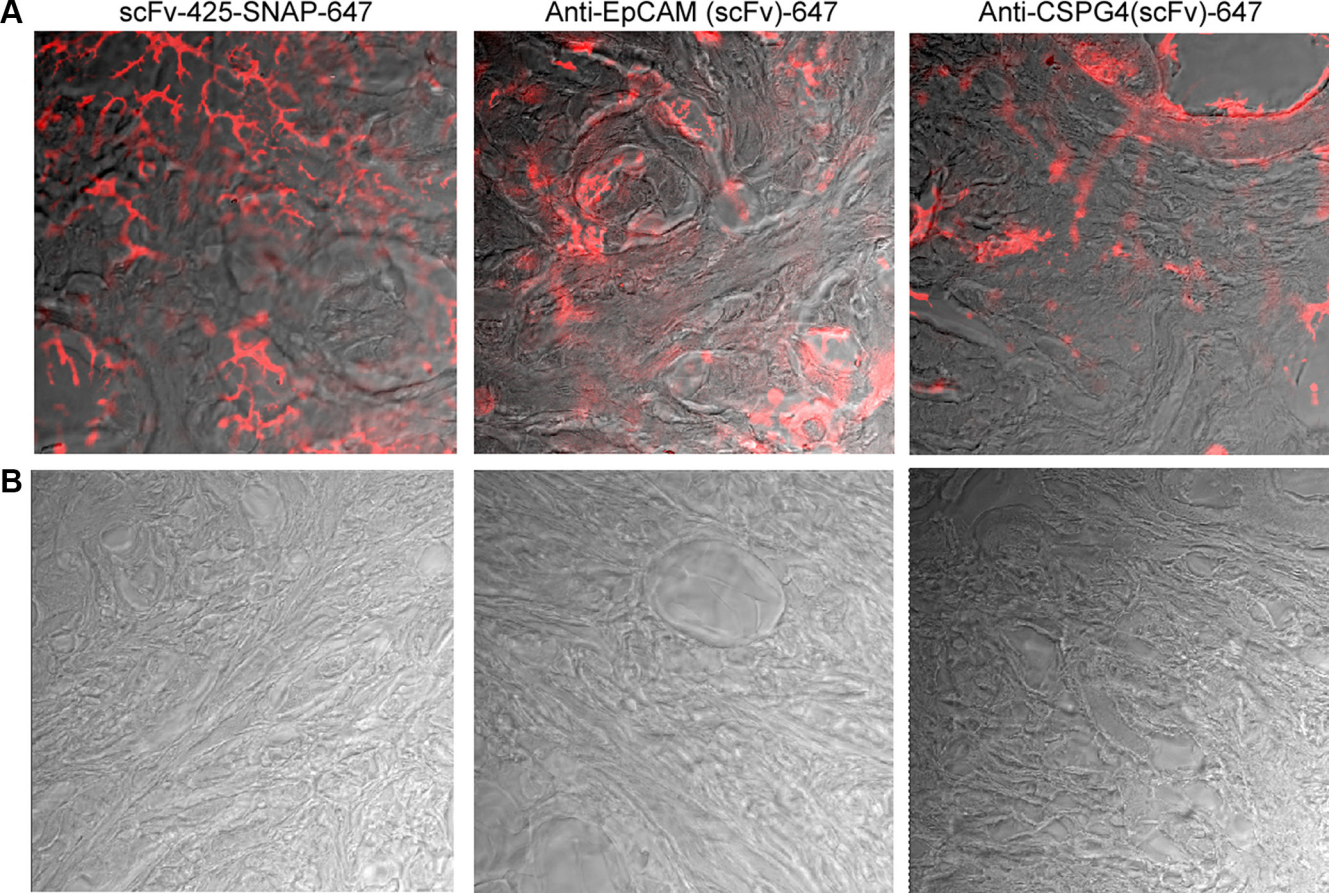
Fluorescence immunohistochemistry of scFv-SNAP-Alexa Fluor^®^647-labeled proteins against EGFR, EpCAM and CSPG4 in human breast cancer tissues (A) and normal breast tissue (B) Biopsies were incubated with scFv-425-SNAP-Alexa Fluor^®^647, anti-EpCam(scFv)-SNAP-Alexa Fluor^®^647 or anti-CSPG4(scFv)-SNAP-Alexa Fluor^®^647 overnight at 4°C. A TCS SP5 confocal microscope was used to visualize Alexa Fluor^®^647 signals (red) on the breast cancer tissues.

To investigate the clinical potential of our fusion proteins in human breast cancer, particularly their ability to distinguish between breast tumors and healthy tissues, fluorescence immunohistochemistry analysis was carried out to determine the binding properties of all Alexa Fluor^®^647-labeled proteins against human breast cancer biopsies and normal breast tissues. We confirmed the specific binding of the labeled proteins to breast cancer tissues, and observed no significant binding to normal human breast tissues (Figure 5).

### Photoimmunotoxicity

**Figure 6:**
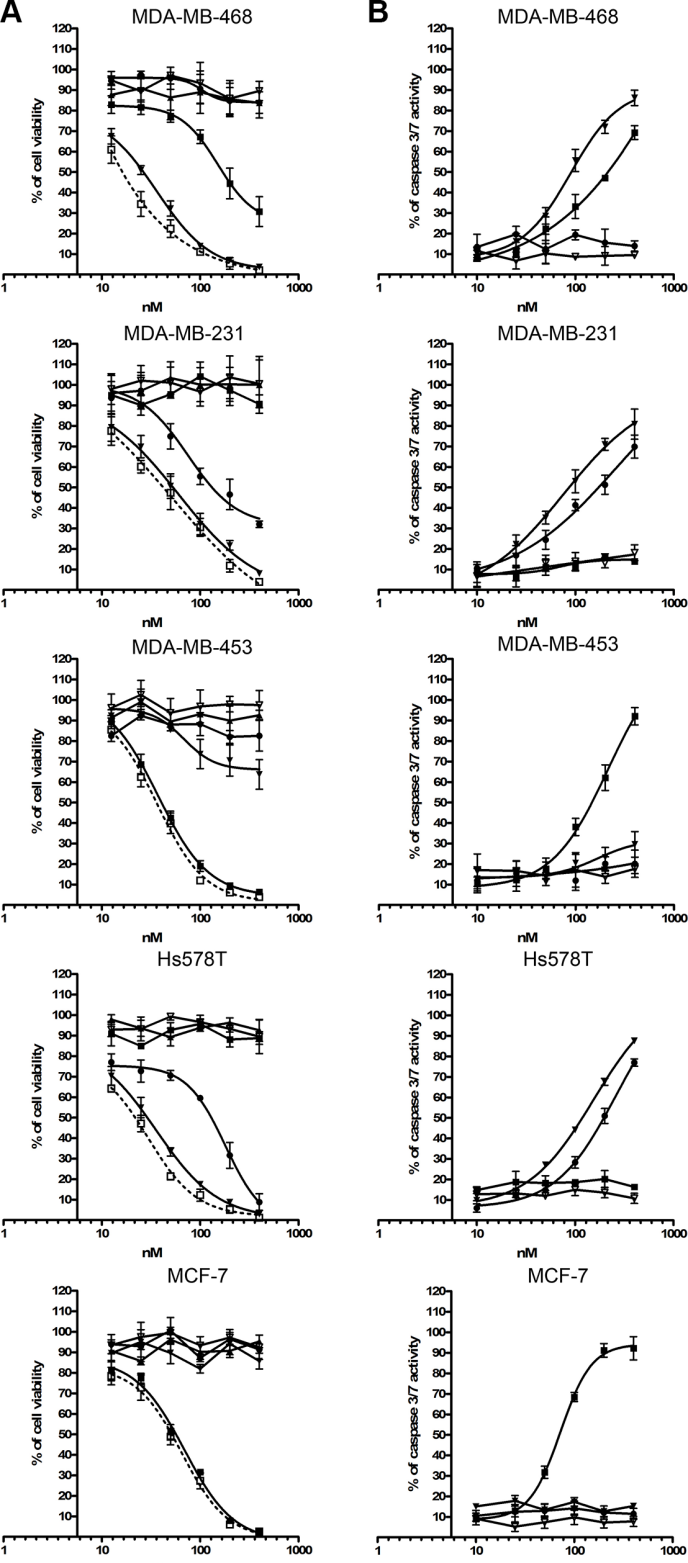
Toxic activity of photoimmunotheranostic agents against four TNBC cell lines and MCF-7 (**A**) The cytotoxicity of scFv-425-SNAP-IR700 (▼), anti-EpCAM(scFv)-SNAP-IR700 (■), anti-CSPG4(scFv)-SNAP-IR700 (●), the mixture of all three photoimmunotheranostic agents (□), IR700 only (Δ) and the mixture of all three photoimmunotheranostic reagents applied in the absence of NIR irradiation (▲) was evaluated by cell viability assays using four different TNBC cell lines (MDA-MB-468, MDA-MB-453, MDA-MB-231 and Hs758T) and one ER^+^ cell line (MCF-7). Cells were incubated with increasing concentrations of each reagent, separately or in combination, or with IR700 (0, 10, 25, 50, 100, 200 and 400 nM). (**B**) Induction of apoptosis by photoimmunotheranostic agents (separately) and by IR700, assessed using the Apo-ONE^TM^ Homogeneous Caspase-3/7 assay.

Cell viability was determined using the formazan dye-based cell proliferation XTT kit II (Roche, Mannheim Germany). Cell viability was reduced in a dose-dependent manner following light treatment after cell incubation with each of the individual photoimmunotheranostic reagents for 24 h at 37°C, corresponding to the expression levels of EGFR, EpCAM and CSPG4. The photoimmunotheranostic reagents were able to eliminate target TNBC cell lines with IC_50_ values of 62–165 nM (Table 3). Furthermore, an equimolar mixture of all three photoimmunotheranostic agents increased the toxicity towards TNBC cells by up to 40% compared to the individual reagents (Table 3). Unconjugated IR700 had no toxic effect against any of the treated cells even under illumination. Also, no toxic effects were observed when the cell lines were incubated with a mixture of all three photoimmunotheranostic agents without NIR light treatment. (Figure 6A).

**Table 3:** Toxicity of scFv-425-SNAP-IR700, anti-EpCAM(scFv)-SNAP-IR700 and anti-CSPG4(scFv)-SNAP-IR700 against different TNBC cell lines and the ER^+^ cell line MCF-7, represented as IC_50_ values (nM)

	scFv-425-SNAP- IR700	Anti-EpCAM (scFv)-SNAP-IR700	Anti-CSPG4 (scFv)-SNAP-IR700	Cocktail reagents
**MDA-MB-468**	26	165	-	18
**MDA-MB-231**	61	-	128	34
**MDA-MB-453**	-	40	-	36
**Hs758T**	29	-	131	18
**MCF-7**	-	56	-	51

Data are presented as means for three replicated experiments.

Furthermore, the ability of the photoimmunotheranostic agents to trigger selective apoptosis was confirmed using the Apo-ONE^TM^ Caspase-3/7 assay. The levels of activated caspase-3 and caspase-7 in treated cells directly correlated with the expression levels of the target receptors (Figure 6B). More importantly, no significant reduction in cell viability and no induction of caspase-3 or caspase-7 was detected in cells treated with the unconjugated IR700 even under illumination (Figure 6B).

## DISCUSSION

Antibody-based tumor-targeting therapies represent a substantial step towards improved cancer management by selectively targeting tumor cells and concomitantly reducing adverse effects [22]. Treatments with antibodies involve one of three mechanisms: receptor blockade, antibody-dependent cellular cytotoxicity, or complement-dependent cytotoxicity. However, multiple high doses of the antibody are required for effective treatment [23].

The efficacy of passive therapeutic antibodies has been improved by covalently linking them to synthetic cytotoxic molecules to yield ADCs, which are now successful for the treatment of several forms of cancer, including CD30^+^ lymphoma and HER2^+^ breast cancer [24, 25].

Most of the cytotoxic components used in ADCs are highly potent membrane-permeable molecules [26], thus off-target toxicity due to premature toxin release into the blood is a major safety challenge in ADC development [27]. One appealing approach to overcome this drawback is the use of photoimmunotheranostic agents, which can simultaneously improve cancer detection and achieve on-demand treatment. Such reagents combine the phototoxic and imaging properties of certain photosensitizers with the specificity of antibodies, allowing the detection and inducible elimination of tumor cells. Photoimmunotheranostic reagents offer a minimally invasive intervention that uses nontoxic photosensitizers that can be activated locally and on-demand using harmless light to produce cytotoxic reactive oxygen species that kill malignant cells by inducing cell apoptosis and/or necrosis [28].

Each of the components of our photoimmunotheranostic agents has minimal toxicity when presented alone, thus toxicity is achieved only when the components are applied together at the appropriate place and time. Consistent with previous studies [29–31], we found that treating cells with one component (i.e. light, the photosensitizer or the antibody) did not reduce cell viability or induce apoptosis. Furthermore, the overall toxicity of the photoimmunotheranostic reagents depended on the presence and copy number of cell surface receptors recognized by the antibody. Unlike other photosensitizers and small cytotoxic agents, free IR700 showed no off-target effects even under illumination, and it only became active following receptor-antibody interactions [30].

The correlation between cytotoxicity and cell surface receptor density combined with the intrinsic safety of IR700 increases the therapeutic potential of our photoimmunotheranostic agents and offers a failsafe system to prevent off-target effects. In this context, a photoimmunotheranostic approach may also help to address the challenge of multidrug resistance in cancer cells, which is the main reason for the failure of several cancer treatments. Following the activation of photosensitizers with a suitable wavelength and duration of light, the energy-enriched photosensitizer releases the extra energy to the neighboring substrates and returns to its ground state [28]. A portion of the excited singlet state molecules is converted through intersystem crossing into the relatively long-lived (micro-to-milliseconds) excited triplet state, which can either generate free radicals or radical ions via hydrogen atom removal or electron transmission to the surrounding biological substrates, solvent molecules or oxygen. This reaction generates *in situ* toxic free radicals or reactive oxygen species, which ultimately cause tumor cells ablation by inducing apoptosis and/or necrosis [28]. Recently, the activation of IR700 with light at 500–780 nm for 1 min was shown to increase water diffusion into treated cells resulting in an expansion of the cell to its maximum size, ultimately damaging the membrane and inducing necrosis [32]. Direct tumor cell ablation by the induction of necrosis can provoke an immune response that leads to the release of inflammatory mediators. This stimulates neutrophils and macrophages that play a long-term role in the elimination of neoplastic cells [28]. These distinct mechanisms to induce cell death benefit the development of therapeutic modalities that can be used to overcome tumor cell resistance against other treatment strategies.

Although we only investigated the *in vitro* imaging potential of the three photoimmunotheranostic agents, we found that all three were able to bind specifically to their target receptors in four TNBC cell lines resulting in their internalization, whereas no fluorescence was detected on the cell lines lacking these receptors. Furthermore, fluorescence immunohistochemistry experiments confirmed that our constructs can distinguish between healthy and TNBC tumor tissues, because the latter express high levels of EGFR, EpCAM or CSPG4. IR700-mAb constructs have recently demonstrated their promising theranostic properties *in vivo* against several types of cancer [29, 30]. Beyond detection and elimination of cancer cells, photoimmunotheranostic agents could also be used to monitor the duration of IR700 fluorescence during surgery, thus providing an immediate assessment of treatment efficacy [33].

Photoimmunotheranostic agents should ideally be homogeneous products with defined DARs, because this is necessary for optimal pharmacokinetic, efficacy and safety profiles. ADCs with variable DARs show inconsistent pharmacokinetic behavior, affinity, toxicity and drug release properties, and site-specific conjugation methods should therefore be used to generate ADCs with improved pharmacokinetic properties, therapeutic indices, and safety profiles [9, 34–36]

Although several methods have been developed for the conjugation of therapeutic agents to antibodies, only two are widely used: the modification of thiol groups in cysteine side chains and the modification of amine groups in lysine side chains. These methods are simple, but they cannot produce homogeneous ADCs or photoimmunotheranostic agents with defined pharmacological and safety profiles [8, 9]. We therefore used the self-labeling SNAP-tag to generate homogeneous and stable photoimmunotheranostic agents. SNAP-tag technology allows the covalent attachment of BG-modified IR700 to different scFv-SNAP-tag fusion proteins by the irreversible coupling of an alkyl group to a cysteine residue in the SNAP-tag protein [37]. This is a simple, rapid, site-specific and reproducible method to equip any protein of interest with various BG-modified molecules, ranging from small molecules such as photosensitizers [13] and fluorophores [15, 38] to large molecules such as drug nanocarriers [14].

The heterogeneity, complexity and clinically aggressive behavior of TNBC is challenging for targeted therapies, which work best with a single and defined molecular target. In most cases, anthracycline- and taxane-based chemotherapy is used for the systemic treatment of TNBC, but chemotherapy with or without other treatment options often has a major impact on patient quality of life. Therefore, it is important to develop new strategies that reduce the impact on patients while retaining therapeutic efficacy. Photoimmunotheranostic agents could help to bridge this gap. It is unlikely that any single targeted therapy will be efficacious in all TNBC patients, so we investigated a cocktail of three photoimmunotheranostic agents targeting some of the most abundant receptors in TNBC cells that are also expressed at minimal levels in healthy tissue. Using SNAP-tag technology, specific antibody fragments targeting EGFR, EpCAM and CSPG4 were conjugated with the highly potent photosensitizer IR700D. These reagents showed powerful imaging properties and highly potent phototherapeutic activity *in vitro*. We demonstrated their favorable effect, individually and in combination, against four different TNBC cell lines that express different levels of EGFR, EpCAM and CSPG4, with IC_50_ values in the nanomolar range. The robust safety profile of these reagents was demonstrated by the lack of toxicity caused by the free IR700 dye, even after irradiation. Our promising photoimmunotheranostic agents should now be tested to determine their activity *in vivo* [18, 30, 32, 33].

## MATERIALS AND METHODS

### Cell lines

The human TNBC cell lines MDA-MB-468 (ATCC^®^HTB-132^TM^), MDA-MB-231 (ATCC^®^HTB-26^TM^), MDA-MB-453 (ATCC^®^HTB-131^TM^), Hs578T (ATCC^®^HTB-126^TM^) and ER^+^ MCF-7 (ATCC^®^HTB-22^TM^) were cultured in RPMI-1640 medium supplemented with 2 mM L-glutamine, 10% (v/v) fetal bovine serum (FBS), and 100 U/mL penicillin−streptomycin. The cells were incubated at 37°C and 5% CO_2_. The medium and additives were purchased from Invitrogen, Darmstadt, Germany.

### Modification of IR700 with a benzylguanine linker

The IR700 N-hydroxysuccinimide ester (IR700) fluorophore (LI-COR Biosciences GmbH, Bad Homburg, Germany) was modified with a benzylguanine linker (BG-PEG_24_-NH_2_) (Covalys Biosciences AG, Witterswil, Switzerland) using the N-hydroxysuccinimide ester-amino group reaction as previously described [39]. Briefly, IR700 was mixed with a two-fold molar excess of BG-PEG_24_-NH_2_ in phosphate-buffered saline (PBS, pH 7.4) and incubated overnight in the dark at room temperature. BG-modified IR700 was analyzed and purified by high-performance liquid chromatography (HPLC) (Water, Eschborn, Germany) using a Prontosil C-18 column (250 × 4.6 mm, 5 μm, 120Å) (Bischoff Chromatography, Leonberg, Germany). Runs were monitored at 286 and 680 nm to visualize BG-linkers, IR700 and BG-modified IR700 molecules. BG-PEG_24_-IR700 analysis and purification was carried out using a 55-min gradient from buffer A (0.1 M triethylammonium acetate, TEAA) to buffer B (70% acetonitrile) at a flow rate of 1 mL/min.

### Protein expression and purification

The scFv-425-SNAP-tag, anti-EpCAM(scFv)-SNAP-tag and anti-CSPG4(scFv)-SNAP-tag fusion proteins were expressed in HEK-293T cells and purified from the supernatant using a Ni-NTA Superflow cartridge (Qiagen, Hilden, Germany) on an ÄKTA FPLC system (GE Healthcare Europe GmbH, Freiburg, Germany) as previously described [12, 13, 40, 41].

### Preparation of the photoimmunotheranostic agents

BG-modified IR700 or BG-modified dyes (BG-Vista Green, BG-Alexa Fluor^®^647) (Covalys Biosciences AG) were conjugated to each scFv-SNAP-tag fusion protein by incubating at a 2:1 molar ratio for 2 h in the dark at room temperature. Unconjugated dyes were removed by size-exclusion chromatography using Zeba Spin Desalting Columns, 7K MWCO (Thermo Fisher Scientific, Waltham, Massachusetts, USA). The site-specific conjugation of BG-PEG_24_-IR700 to the SNAP-tag was confirmed by blocking the fusion proteins with bromothenylpteridine (BTP) (Covalys Biosciences AG) or by post-incubating them with BG-Vista Green fluorescent dye. The fluorescence signals of labeled proteins were visualized using the CRi Maestro imaging system (CRi, Woburn, Massachusetts, USA) after protein separation by SDS-PAGE. Protein labeling efficiency was estimated by photometry using the extinction coefficients of the fluorescence dyes and the theoretical extinction coefficient of the proteins.

The stability of each scFv-SNAP-tag fusion protein in serum was estimated by conjugation with IR700 dye as described above followed by incubation in mouse serum for 0, 0.5, 1, 2, 3, 4, 5 and 6 h at 37°C. After protein separation by SDS-PAGE, the IR700 fluorescence signal was visualized using the CRi Maestro imaging system. Thereafter, SDS-PAGE gels were stained with Coomassie Brilliant Blue and advanced image data analyzer (AIDA) software was used to measure the fluorescent signal and Coomassie dye staining intensity.

### Flow cytometry

Although the IR700 dye has advantageous optical properties for *in vivo* imaging, traditional *in vitro* imaging devices based on flow cytometry and confocal microscopy fail to detect its narrow NIR fluorescence peak. To overcome this limitation, BG-Alexa Fluor^®^647 (Covalys Biosciences AG) was conjugated to the fusion protein instead of BG-IR700 for flow cytometry and confocal microscopy.

The binding of the labeled scFv-SNAP fusion proteins to TNBC cell lines expressing EGFR, EpCAM and CSPG4 (MDA-MB-468, MDA-MB-453, MDA-MB-231 and Hs758T) and the ER^+^ breast cancer cell line (MCF- 7) was monitored by flow cytometry using a FACSCalibur device and CellQuest software (Becton & Dickinson, Heidelberg, Germany) after incubating 4 × 10^5^ cells with 0.5 μg of labeled protein in 200 μL PBS for 15 min on ice.

### Internalization assay

Protein internalization was measured by confocal microscopy. MDA-MB-468, MDA-MB-453, MDA-MB-231, Hs758T and MCF-7 cells were seeded in 96- well plates suitable for fluorescence microscopy (Greiner Bio-One, Frickenhausen, Germany) to a density of 4000 cells/well and incubated overnight at 37°C. The cells were incubated with the fluorescent scFv-SNAP-Alexa Fluor^®^647 fusion proteins specific for EGFR, EpCAM and CSPG4 for 60 min at 37°C. Hoechst 33342 fluorescent nuclear counterstain (Thermo Fisher Scientific, Darmstadt, Germany) was added to the cells and incubated for 20 min at 37°C. Images of the cells were captured using the Opera High Content Screening System (PerkinElmer, Rodgau, Germany) with a 40× air objective.

### Human patient samples

Human breast cancer biopsies were provided by the RWTH Aachen University Centralized Biomaterial Bank (cBMB) according to its regulations, following RWTH Aachen University, Medical Faculty Ethics Committee approval (decision EK 206/09).

### Immunofluorescence analysis of human patient samples

The binding properties of the three scFv-SNAP-Alexa Fluor^®^647 fusion proteins against human breast cancer tissues and normal breast tissues were visualized with a TCS SP5 confocal microscope (Leica Microsystems, Wetzlar, Germany). Human breast biopsies were cut into 8–12 μm sections on a Leica CM 3050 cryostat and mounted on coated slides. After drying for 72 h, sections were fixed for 10 min in dry acetone, air-dried then incubated with 2 μg of each scFv-SNAP-Alexa Fluor^®^647 fusion protein in PBS overnight at 4°C. Slides were washed three times with PBS, dried and mounted with Vectashield mounting medium and cover slips.

### Phototoxicity of scFv-425-SNAP-IR700

The phototoxicity of the scFv-SNAP-IR700 proteins and free IR700 was determined using Cell Proliferation (XTT) Kit II (Roche, Mannheim Germany) as previously described [13]. Briefly, MDA-MB-468, MDA-MB-453, MDA-MB-231, Hs758T and MCF-7 cells were seeded in 96-well plates and incubated overnight at 37°C. The cells were incubated with increasing concentrations (0, 10, 25, 50, 100, 200 and 400 nM) of the scFv-SNAP-IR700 fusion proteins or an equivalent concentration of IR700 for 3 h at 37°C in the dark. As a toxic control, cells were incubated with 500 μg/mL Zeocin. After washing the cells three times with PBS, they were incubated in fresh phenol red-free culture medium and irradiated with visible light (VIS) plus water-filtered infrared light A (wIRA) using a Hydrosun Typ 750 radiator with a water-containing cuvette and orange filter OG590, with a spectrum in the range 580−1400 nm (Hydrosun Medizintechnik GmbH, Müllheim, Germany). The irradiation experiments were carried out according to the physical and photobiological laws described by Piazena and Kelleher [42]. The cells were irradiated at 140 mW/cm^2^, at a dose of 25 J/cm^2^ VIS or 76 J/cm^2^ wIRA, for an exposure time of 15 min. During irradiation, the cells were incubated in a temperature-controlled water-bath. Finally, the cells were incubated in fresh medium as described above for another 24 h.

To determine cell viability, cells were incubated with 50 μL of 2,3-bis-(2-methoxy-4-nitro-5-sulfophenyl)-2H-tetrazolium-5-carboxanilide (XTT) reagents for 4 h at 37°C and the reduction of XTT to formazan was monitored by spectrophotometry at 450 nm absorbance wavelength and 630 nm reference wavelength using an ELx808 microplate reader (BioTek Instruments GmbH, Bad Friedrichshall, Germany).

To verify the ability of photoimmunotheranostic agents to induce apoptosis, the activities of caspase-3 and caspase-7 were analyzed in cell lysates using the Apo-ONE^®^ Homogeneous Caspase-3/7 assay (Promega, Mannheim, Germany). After treating the cells as described above, they were incubated with 100 μL of Apo-ONE reagent for 6 h, then the fluorescence signal was measured using the ELx808 microplate reader with an excitation wavelength of 485 nm and an emission wavelength of 535 nm.

### Data analysis

Statistical analysis was carried out using GraphPad Prism v5.00 for Windows (GraphPad Software, San Diego, California, USA). Data are presented as means ± SEM for at least three replicated experiments.
